# Effectiveness of behavioural economics-based interventions to improve colorectal cancer screening participation: A rapid systematic review of randomised controlled trials

**DOI:** 10.1016/j.pmedr.2022.101747

**Published:** 2022-03-03

**Authors:** Lily C. Taylor, Robert S. Kerrison, Benedikt Herrmann, Sandro T. Stoffel

**Affiliations:** a The Primary Care Unit, Department of Public Health and Primary Care, University of Cambridge, UK; b Research Department of Behavioural Science and Health, University College London, London, UK; c School of Health Sciences, University of Surrey, Surrey, UK; d European Commission, Joint Research Centre (JRC), Ispra, Italy; e Institute for Pharmaceutical Medicine, University of Basel, Basel, Switzerland

**Keywords:** Colorectal cancer screening, Behavioural economics, RCT, MINDSPACE, Systematic review

## Abstract

• We searched PubMed, PsycInfo and EconLit for RCTs that evaluated BE interventions in CRC screening.
• We identified 1027 papers for title and abstract review. 30 studies were eligible for the review.
• The most frequently tested BE intervention was incentives, followed by default principle and salience.
• Default-based interventions were most likely to be effective. Incentives had mixed evidence.
• BE remains a promising field of interest in relation to influencing CRC screening behaviours.

## 1 Introduction

### 1.1 Background

Colorectal cancer (CRC) is a leading cause of death in Europe, Asia and North America (Bowel cancer, 2017, Schreuders et al., 2015, Sung et al., 2021). Survival is greatly improved when CRC is diagnosed early (Bowel cancer statistics, 2015, Koo et al., 2017) and, as a result, many countries throughout the world have implemented organised screening programmes for the early detection of CRC (Schreuders et al., 2015).

The most commonly used tests for CRC screening programmes are home-based stool tests (e.g. the ‘guaiac faecal occult blood test’ [gFOBT] and the ‘faecal immunochemical test’ [FIT]) (Schreuders et al., 2015, Koo et al., 2017, Li et al., 2021), followed by endoscopic techniques (e.g. colonoscopy, flexible sigmoidoscopy [FS]) and scans (e.g. CT Colonograph) (Schreuders et al., 2015).

Despite the availability of several effective CRC screening tests, uptake is often low compared with other organised screening programmes, such as those for breast and cervical screening (Koo et al., 2017). Participation rates routinely fall short of national and international guidelines (e.g. the European Guidelines for Quality Assurance in Colorectal Cancer Screening and Diagnosis, which has a minimum participation rate of 65%) (Koo et al., 2017, Duffy et al., 2017, Directorate-General for Health and Consumers, 2010). As a result, there is currently much interest in the development of effective interventions to improve CRC screening uptake.

Historically, a wide range of interventions to improve CRC screening uptake have been investigated, including advance notification letters (also referred to as: ‘pre-screening reminders’), mailed reminders and telephone navigation calls (Duffy et al., 2017). Such strategies have focussed on a cognitive model of behaviour change, aiming to influence behaviour via reflective, rational thought processes known as System II thinking (Dolan et al., 2012).

More recently, researchers have begun testing interventions grounded in behavioural economics (BE). Such interventions seek to exploit the automatic (learned) responses of System I thinking, called ‘heuristics’ (Dolan et al., 2012, Purnell et al., 2015, The rise of behavioural economics, 2017), and usually involve small changes, known as ‘nudges’, to the context in which the decision is being made (Thaler and Sunstein, 2009).

Several types of nudge have been described, each of which can be leveraged differently to try and improve CRC screening participation (Purnell et al., 2015). Choice architecture, for example, refers to the way in which choices are presented, and can be optimised to encourage participation for certain test options (Purnell et al., 2015). Similarly, perceived social norms, default options, financial incentives and other principles can all be leveraged to unconsciously influence screening behaviours in a positive manner (Purnell et al., 2015).

While the concept of BE has gained traction over the past two decades, it is still a relatively new and emerging field in relation to cancer screening, and CRC screening more specifically (Dolan et al., 2012). Indeed, to date, the evidence base for BE interventions, in relation to CRC screening, remains uncertain, with no formal review of the literature having been published. Previous studies have explored some BE-related interventions, such as GP endorsement (Duffy et al., 2017) and incentives (Facciorusso et al., 2021); however, none has attempted to perform a review of the plethora of BE interventions that exist.

The present study, therefore, seeks to address this dearth in the literature, by synthesising data from existing studies to investigate whether interventions using BE principles are effective at influencing CRC screening behaviours in individuals eligible for population-based screening.

## 2 Methods

### 2.1 Search strategy and study design

We searched PubMed (May 2021) for randomised controlled trials (RCTs) that tested the effectiveness of BE interventions to improve participation in CRC screening. To be eligible, a full text English article had to be available. Studies were excluded if they did not use a RCT design, or measured intentions instead of behaviour (see Table 1 for a detailed overview of the inclusion / exclusion eligibility criteria).

**Table 1:** Eligibility criteria.

**Inclusion criteria:**	**Exclusion criteria:**
Published and peer reviewed	Full text unavailable
English language	Non-English language
RCT	Online experiments, observational or qualitative study design
Specific to CRC screening	Symptomatic or surveillance pathways
Informed by BE	Patient education, decision aid or navigation study
Effect of BE component extricable from any multi-faceted interventions	Trial not yet completed

Due to resource restrictions (e.g. funding, staff, etc.), a rapid review of the literature was performed. Therefore, rather than running a single search with the full, comprehensive list of search terms, we performed an initial search, using a narrow selection of search terms (guided by the PICOS framework – see Table 2). The string was then expanded, successively, by adding a small number of additional search terms (to each PICOS component), using the Boolean operator ‘OR’, from a pool of search terms identified from the previous literature (Duffy et al., 2017, Purnell et al., 2015).

**Table 2:** PICOS eligibility criteria.

**Population:**	Any individual eligible for organised screening within the population in question.
**Interventions:**	CRC screening interventions utilising BE principles.
**Comparator:**	Any other intervention targeting CRC screening behaviours.
**Outcomes:**	Participation in CRC screening. Tangible behaviour change, not intentions.
**Study design:**	Randomised controlled trials.

The exact combination and order in which search terms were added to the search string were determined by running multiple searches in PubMed, with the combination providing the largest number of results being the one selected for the expansion at each stage (for transparency, the individual searches and number of results received for each is available from Open Science Framework: https://osf.io/tqsmc/).

After each expansion, title and abstract review was performed for the combination that received the most results. This process of identifying the optimal combination of search terms, expanding the search string, and performing title and abstract review was continued until no new publications were eligible upon title and abstract review. At this point, it was assumed that further expansion of the search string was unlikely to yield additional publications eligible for inclusion.

To minimise the risk of excluding eligible studies not available on PubMed, the final search was also performed on PsycInfo and EconLit (see Table 3).

**Table 3:** Results of the search string expansion in PubMed.

**PubMed Search String:**	**Number of Publications:**	**Number of publications selected by one or two reviewers on title review:**	**Number of publications selected by one or two reviewers on abstract review:**	**% of publications eligible:**
**Search 1:** ((Bowel cancer OR colorectal cancer OR faecal immunochemical test* OR faecal occult blood test* OR colonoscopy OR flexible sigmoidoscopy OR colonography) AND (screen*) AND (behavioural economics OR behavioral economics OR nudg* OR messenger OR incentiv* OR norms OR default* OR salience OR priming OR affect OR commitment OR ego) AND (behaviour OR behavior OR participation OR adherence OR uptake) AND (randomised controlled trial OR randomized controlled trial OR field experiment OR randomised trial OR randomized trial OR RCT OR controlled trial))	175	40	25	14%
**Search 2:** ((Bowel cancer OR colorectal cancer OR faecal immunochemical test* OR faecal occult blood test* OR colonoscopy OR flexible sigmoidoscopy OR colonography OR FIT OR FOBT OR gFOBT OR FS) AND (screen*) AND (behavioural economics OR behavioral economics OR nudg* OR messenger OR incentiv* OR norms OR default* OR salience OR priming OR affect OR commitment OR ego OR heuristics OR bias OR aversion OR decision fatigue OR regret) AND (behaviour OR behavior OR participation OR adherence OR uptake OR utilisation OR utilization) AND (randomised controlled trial OR randomized controlled trial OR field experiment OR randomised trial OR randomized trial OR RCT OR controlled trial))	231	12	2	<1%
**Search 3:** ((Bowel cancer OR colorectal cancer OR faecal immunochemical test* OR faecal occult blood test* OR colonoscopy OR flexible sigmoidoscopy OR colonography OR FIT OR FOBT OR gFOBT OR FS OR colon cancer OR rectal cancer) AND (screen*) AND (behavioural economics OR behavioral economics OR nudg* OR messenger OR incentiv* OR norms OR default* OR salience OR priming OR affect OR commitment OR ego OR heuristics OR bias OR aversion OR decision fatigue OR regret OR order effect*) AND (behaviour OR behavior OR participation OR adherence OR uptake OR utilisation OR utilization OR practices) AND (randomised controlled trial OR randomized controlled trial OR field experiment OR randomised trial OR randomized trial OR RCT OR controlled trial))	113	2	0	0

### 2.2 Study selection process

Title and abstract reviews were performed by two reviewers (LT and RK). Each reviewer assigned publications a value of 1 (‘include’) or 0 (‘exclude’). Any paper that received a score of 1, from either reviewer, following title and abstract review, underwent full paper review. As with title and abstract review, two reviewers assigned the full text of publications a value of 1 (‘include’) or 0 (‘exclude). Unlike title and abstract review, however, disagreements regarding full-text review were resolved through discussion with a neutral arbitrator (SS).

Finally, the reference lists of publications that passed full paper review were searched for further publications (as well as a relevant review published by Duffy and colleagues (2017); (Duffy et al., 2017) not available on PubMed, PsycInfo and EconLit. As with the database searches, publications reported in the reference lists underwent title, abstract and full paper review by two authors (with disagreements resolved through discussion with a third during full paper review).

### 2.3 Data extraction

Data on the author, year of publication, title, primary aim, intervention delivered, method of randomisation (individual or cluster), population characteristics (e.g. age, sex, ethnicity, etc.), timepoint at which participation in screening was measured and the statistical method used (univariate or multivariate) was extracted for each study using customised Excel templates.

### 2.4 Data categorisation

As the field of BE contains many synonymous terms for the different BE principles (for example the term ‘choice architecture’ may be used to describe multiple types of intervention), RCTs were categorised according to the type of BE intervention tested, using the popular MINDSPACE framework (Dolan et al., 2012). MINDSPACE is a mnemonic framework comprised of nine behavioural science principles that can be used to influence behaviour through automatic processes, as shown in Table 4 (Dolan et al., 2012). It was developed specifically to aid in policy making and is, therefore, relevant to the real-life application of this research (MINDSPACE.pdf, 2021). Studies, that tested a combination of interventions, were categories according to their primary BE intervention. Note that in the results section the categories are presented in order of number of studies found and not the order of Table 4.

**Table 4:** Summary of the components included in the MINDSPACE behavioural science framework.

**Messenger:**	The source of the information being communicated to us influences our automatic reaction. This may depend on the level of authority the messenger possesses, e.g. GP endorsement of CRC screening (Dolan et al., 2012, Purnell et al., 2015).
**Incentives:**	Financial incentives and vouchers can be used in various ways to encourage CRC screening uptake. Predictable heuristics dictate how we are likely to respond to the size and timing of incentivisation, e.g. using loss-framing as opposed to equivalent gains (Dolan et al., 2012).
**Norms:**	How others behave can influence individual behaviour through the concept of sociocultural norms (Dolan et al., 2012). Descriptive norms which highlight the behaviour of others and injunctive norms, highlighting what others believe one ought to do, have both been used to examine cancer-related behaviours (Smith-McLallen and Fishbein, 2008).
**Defaults:**	Structuring screening invitations such that the default represents the most beneficial option can improve associated behaviours, as individuals often resort to default options over active choices, e.g. opt-out instead of opt-in screening (Dolan et al., 2012).
**Salience:**	The most relevant information is generally what attracts our attention. Therefore, increasing the salience of CRC in relation to an individual’s personal circumstances may improve related behaviour (Dolan et al., 2012).
**Priming:**	Priming refers to pre-activation of knowledge with cues that may unconsciously impact subsequent behaviour (Dolan et al., 2012), e.g. asking a certain question prior to screening invitation may impact the response.
**Affect:**	Emotional responses are often automatic and may be acted upon before rational decision making occurs (Dolan et al., 2012, Purnell et al., 2015).
**Commitment:**	The conscious act of pre-commitment to a behaviour may subconsciously improve ensuing behaviours, as people strive to deliver on public commitments (Dolan et al., 2012). In CRC screening, asking for commitment such as pledging could be used to increase the likelihood of participation.
**Ego:**	The notion of self-image may motivate individuals to act in ways that facilitate positive self-evaluation (Dolan et al., 2012).

### 2.5 Data analysis

Descriptive statistics were used to quantify study characteristics and the number and percentage of RCTs that reported a positive effect, negative effect, or no effect of BE interventions on CRC screening participation (collectively and stratified by MINDSPACE component). No meta-analysis was conducted due to the high degree of overlaps between different components and differences in mode of employment. Furthermore, according to the Cochrane Rapid Reviews Methods Group, meta-analysis should only be conducted where studies are able to be appropriately pooled (Garritty et al., 2021).

### 2.6 Risk of bias

Eligible papers were assessed for bias using version 2 of the Cochrane risk-of-bias tool for randomised trials (RoB 2) (RoB 2, 2021). A test version of the RoB 2 tool for cluster-randomised (Inadomi et al., 2012, Tinmouth et al., 2015, Nisa et al., 2019, Kullgren et al., 2014, Dacus et al., 2018, Raine et al., 2016, Cole et al., 2002, Zajac et al., 2010, Stoffel et al., 2019, Coronado et al., 2020, Raine et al., 2016, Stoffel et al., 2021a) trials or crossover trials (Slater et al., 2018, Van Roosbroeck et al., 2012) was used where appropriate. RoB 2 is recommended when assessing bias in randomised trials and consists of signalling questions around several domains where bias can occur, including randomisation process, missing outcome data, and reporting of results (RoB 2, 2021). Trials can be classified as ‘low risk’ of bias, ‘high risk’ of bias or raising ‘some concerns’ (RoB 2, 2021). This was conducted by the primary reviewer (LT) with a secondary reviewer (SS) completing risk of bias assessment for 20% of studies.

### 2.7 Transparency

The review was registered prospectively with PROSPERO (reference number: CRD42021253534) and written in accordance with PRISMA guidelines (see Appendix A for completed checklist).

## 3 Results

### 3.1 Search results

**Fig. 1:**
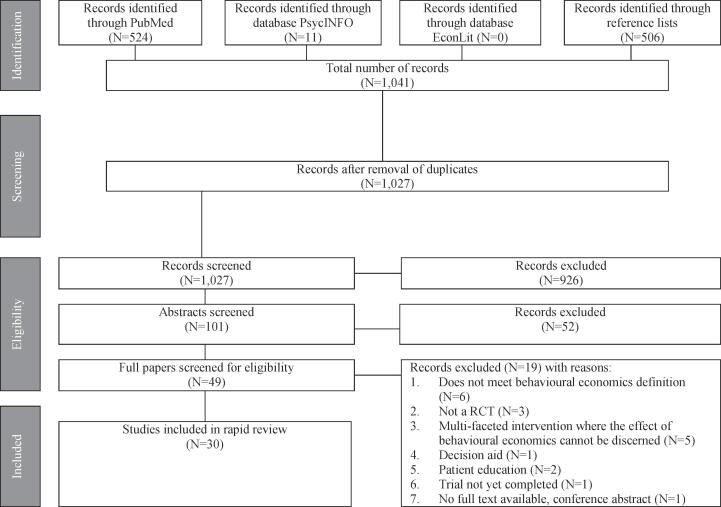
Flow diagram of search results.

In total, the database and reference list searches identified 1041 articles (524 from PubMed, 11 from PsycInfo, 0 from EconLit [confirmed to be a ‘true zero’ by searching the database for eligible papers identified through PubMed and PsycInfo] and 506 from the reference lists). After removing 14 duplicates, 1027 papers were eligible for title and abstract review, of which 49 passed and underwent full paper review. A total of 29 studies were deemed eligible and were included in the review. This number was increased to 30, following the inclusion of an additional paper, authored by RK and SS (Stoffel et al., 2021a), which was accepted for publication, but not published, after the database searches and reference list searches were performed. An overview of the search results is provided in Fig. 1.

### 3.2 Study characteristics

Table 5 presents a summary of the studies included in the review (a detailed overview of each study is provided in Table 6). The majority of studies were conducted in the USA (N = 17, 57%) (Inadomi et al., 2012, Kullgren et al., 2014, Dacus et al., 2018, Slater et al., 2018, Mehta et al., 2018, Church et al., 2004, Huf et al., 2021, Bakr et al., 2020, Mehta et al., 2019a, Mehta et al., 2019b, Mehta et al., 2020a, Mehta et al., 2020b, Lieberman et al., 2019, Green et al., 2019, Mehta et al., 2017, Gupta et al., 2016), examined the effectiveness of BE interventions to promote the uptake of gFOBT or FIT (N = 27, 90%) and had a sample size of ≤ 5,000 participants.

**Table 5:** Summary of study design features.

Design feature:		Number of studies:	References
Country:	United States	17 (57%)	Bakr et al., 2020, Church et al., 2004, Coronado et al., 2020, Dacus et al., 2018; Green et al., 2019, Gupta et al., 2016, Huf et al., 2021, Inadomi et al., 2012; (Kullgren et al., 2014); Lieberman et al., 2019, Mehta et al., 2017, Mehta et al., 2018, Mehta et al., 2019a, Mehta et al., 2019b, Mehta et al., 2020a, Mehta et al., 2020b, Slater et al., 2018
	Australia	3 (10%)	(Cole et al., 2002); MACS group; 2006; (Zajac et al., 2010).
	Canada	1 (3%)	(Tinmouth et al., 2015).
	Belgium	1 (3%)	Van Roosbroeck, Hoeck & Van Hal, 2012.
	Scotland, UK	1 (3%)	O’Carroll et al., 2015.
	Malta	1 (3%)	Stoffel et al., 2021a
	Portugal	1 (3%)	Nisa et al., 2019.
	England, UK	2 (7%)	(Raine et al., 2016). (Raine et al., 2016).
	Spain	1 (3%)	(Stoffel et al., 2019).
	Israel	1 (3%)	Hagoel et al., 2016.
	Cyprus	1 (3%)	Stoffel et al., 2021b.
Screening test(s):	Colonoscopy	9 (30%)	Dacus et al., 2018, Green et al., 2019, Inadomi et al., 2012; MACS group, 2006; Mehta et al., 2017, Mehta et al., 2019a, Mehta et al., 2020a, Slater et al., 2018.
	FIT	13 (43%)	Bakr et al., 2020, Coronado et al., 2020, Green et al., 2019, Gupta et al., 2016, Huf et al., 2021, Lieberman et al., 2019, Mehta et al., 2018, Mehta et al., 2019a, Mehta et al., 2019b, Mehta et al., 2020b, Stoffel et al., 2021a, Stoffel et al., 2021b, Van Roosbroeck et al., 2012
	FOBT	14 (47%)	Church et al., 2004; (Cole et al., 2002); Dacus et al.; 2018; Hagoel et al., 2016, Inadomi et al., 2012; (Kullgren et al., 2014); MACS group; 2006; Nisa et al., 2019, O’Carroll et al., 2015; (Raine et al., 2016, Raine et al., 2016); Stoffel et al.; 2021a; (Tinmouth et al., 2015); Zajac et L.; 201
	CTC	2 (7%)	Bakr et al., 2020; MACS group, 2006
	FS	4 (13%)	Bakr et al., 2020, Dacus et al., 2018; Green et al., 2019; MACS group, 2006
Sample size:	N = 200–5000	19 (63%)	Bakr et al., 2020, Church et al., 2004; (Cole et al., 2002); Coronado et al., 2020, Green et al., 2019, Huf et al., 2021, Inadomi et al., 2012; MACS group, 2006; Mehta et al., 2017, Mehta et al., 2018, Mehta et al., 2019a, Mehta et al., 2019b; Mehta et a., 2020a; Mehta et al., 2020b, Nisa et al., 2019; (Kullgren et al., 2014); Stoffel et al.; 2021b; (Tinmouth et al., 2015, Zajac et al., 2010)
	N = 5001–10,000	4 (13%)	Gupta et al., 2016, Lieberman et al., 2019; (Stoffel et al., 2019); Stoffel et al.; 2021a
	N = 10,001–50,000	3 (10%)	Dacus et al., 2018, Hagoel et al., 2016, Van Roosbroeck et al., 2012
	N = 50,001–100,000	2 (7%)	O’Carroll et al., 2015. Slater et al., 2018.
	N > 100,000	2 (7%)	(Raine et al., 2016). (Raine et al., 2016).
RoB 2 score:	Low risk	12 (40%)	Coronado et al., 2020, Green et al., 2019, Huf et al., 2021; (Kullgren et al., 2014); Mehta et al., 2017, Mehta et al., 2018, Mehta et al., 2019a, Mehta et al., 2019b, Mehta et al., 2020a, Mehta et al., 2020b, O’Carroll et al., 2015, Slater et al., 2018
	Some concern	16 (53%)	Bakr et al., 2020; (Cole et al., 2002, Dacus et al., 2018); Gupta et al., 2016, Hagoel et al., 2016, Inadomi et al., 2012, Lieberman et al., 2019; MACS group, 2006; Nisa et al., 2019; (Raine et al., 2016, Raine et al., 2016, Stoffel et al., 2019); Stoffel et al., 2021a, Stoffel et al., 2021b; Tinmouth et al., 2014; Van Roosbroeck, Hoeck & Van Hal, 2012.
	High risk	2 (7%)	Church et al., 2004; (Zajac et al., 2010).

**Table 6:** Summary of study characteristics, experimental conditionals, and findings.

Author (year):	Country:	Screening test:	Intervention type:	Sample size:	Experimental conditions:	Findings:
(Cole et al., 2002)	Australia	FOBT	Messenger	2,400	Control: standard invite. Arm 1: invitation indicating GP practice support. Arm 2: invitation on GP practice letterhead, signed by practice partner.	Arm 1 & Arm 2 differed significantly from control OR 0.77 (95% CI: 0.60–0.98). Arm 2 showed trend of higher participation compared to arm 1 but was not significant.
(Zajac et al., 2010)	Australia	FOBT	Messenger	1,800	Control: invitation without endorsement. Arm 1: invitation indicating GP practice support. Arm 2: invitation on practice letterhead, signed by GP.	Arm 1 & arm 2 had significantly enhanced participation across all 4 screening rounds compared to control. Arm 2 participants were not more likely to participate than arm 1. Participation in control ER group: 33%, 37%, 40% & 36%, arm 1: 39%, 42%, 45% & 44%, arm 2: 42%, 47%, 48% & 49%.
(Kullgren et al., 2014)	United States	FOBT	Incentive	1,549	Stage 1: Control: usual care. Arm 1: $5 incentive. Arm 2: $10 incentive. Arm 3: $20 incentive. Stage 2: Control: usual care. Arm 1: $5 incentive. Arm 2: 1in10 chance of $50. Arm 3: entry into $500 raffle.	Stage 1: None of the incentives increased rate of FOBT completion. Stage 2:1in10 chance of $50 increased completion vs usual care (difference 19.6%, *P* <.001), $5 incentive or $500 raffle did not. Only 1in10 chance of receiving $50 was effective.
Gupta et al., 2016	United States	FIT	Incentive	8,565	Control: mailed outreach. Arm 2: mailed outreach plus $5 incentive upon FIT completion. Arm 3: mailed outreach plus $10 upon FIT completion.	No significant difference in FIT uptake for any incentive (36.9%) vs no incentive (36.2%).No significant difference for the $10 incentive (34.6%) or the $5 incentive (39.2%) individually. Trend for higher completion in the incentive vs no incentive group but not statistically significant (control: 57%, $5: 63%, $10: 62%, *P* = 0.40).
Mehta et al., 2017	United States	Colonoscopy	Incentive	2,245	Control: email with phone no. to schedule colonoscopy. Arm 1: active choice: email with choice to opt-in or out of colonoscopy. Arm 2: financial incentive: active choice plus $100 conditional incentive.	No statistical difference in colonoscopy completion between control (1.6%) & active choice (1.5%). Financial incentive arm had higher completion rate (3.7%) than both arms (*P* =.011).Greater proportion of participants said yes to screening in the financial incentive arm compared to active choice (15.5% vs 8.7%, *P* < 0.01).
(Dacus et al., 2018)	United States	FOBT, sigmoidoscopy, colonoscopy	Incentive	18,066	Control: no mailed reminder, usual care. Arm 1: mailed reminder. Arm 2: mailed reminder plus $25 cash card incentive.	Adirondack region: No mailed reminder: 5.8%, mailed reminder: 7.0%, mailed reminder & incentive: 7.2% Central region: No reminder: 6.5%, mailed reminder: 7.2%, mailed reminder & incentive: 6.9% Neither region had statistically significant differences.
Slater et al., 2018	United States	Colonoscopy	Incentive	94,294	Control: usual care, received intervention 15 months later. Arm 1: direct mail materials & $20 incentive, patient navigation phone no.	Odds of receiving a colonoscopy were significantly higher in arm 1 than in the control (OR = 1.12; 95% CI = 1.04–1.21). Odds of being screened in the intervention arm increased by 12% relative to control.
Green et al., 2019	United States	FIT, FS, colonoscopy	Incentive	898	Arm 1: mail only, <3 mailings with screening info & test choices, FIT kit & reminder. Arm 2: mailings plus $10 upon screening completion. Arm 3: mailings plus 1in10 chance of receiving $50 upon screening completion.	Completion of any screening was not significantly higher for monetary or lottery groups than for mail only (*P* = 0.11).Interventions had a significant effect (*P* = 0.04) on FIT completion with net increase of 7.7% (95% CI 0.3–15.1%) for mail & monetary group, & 7.1% (95% CI: −0.2–14.3%) in the mail & lottery group, compared to mail only.
Lieberman et al., 2019	United States	FIT	Incentive	8,565	Arm 1: outreach only. Arm 2: outreach plus $5 incentive for FIT return. Arm 3: outreach plus $10 incentive for FIT return.	Year 1 FIT completion: 36.9% with incentives vs. 36.2% outreach alone (*P* = 0.59), not statistically different for $10 (34.6%; *P* = 0.31) or $5 (39.2%; *P* = 0.07) vs. outreach alone. Year 2 completion: 61.6% with incentives vs. 60.8% outreach alone (*P* = 0.75), not statistically different for $10 or $5 vs. outreach alone. Year 3 completion: 79.4% with incentives vs. 74.8% outreach alone (*P* = 0.08), higher for $10 (82.4%) vs. outreach alone (*P* = 0.033), but not for $5 vs. outreach alone.
Mehta et al., 2019b	United States	FIT	Incentive	897	Control: no financial incentive. Arm 1: unconditional $10 incentive included with the mailing. Arm 2: conditional $10 incentive upon FIT completion. Arm 3: conditional lottery with 1in10 chance of winning $100 after FIT completion.	Completion rate at 2 months: 26.0% (95% CI, 20.4–32.3%) in control, 27.2% (95% CI, 21.5–33.6%) in arm 1 23.2% (95% CI, 17.9–29.3%) in arm 2 & 17.7% (95% CI, 13.0–23.3%) in arm 3. None incentivises were statistically superior to the control.No significant difference at 6 months.
Nisa et al., 2019	Portugal	FOBT	Incentive	1,652	Arm 1: single €10 incentive upon screening completion. Arm 2: two €5 incentives at beginning & end of screening.	93.2% of people who collected the kit completed screening in arm 1 & 97.7% in arm 2.Completion rate of 61.1% in arm 2 vs 41.4% in arm 1 (*P* <.001). Compared with single incentive, likelihood of those offered partitioned incentives completing screening & delivering their results more than doubled (OR = 2.24, 95% CI: 1.84–2.71).
Mehta et al., 2020a	United States	Colonoscopy	Incentive	1,172	Control: web-based risk assessment & colonoscopy scheduling. Arm 1: control plus $10 loss-framed incentive to complete risk assessment, additional $25 unconditional incentive for colonoscopy completion.	Incentive increased risk assessment participation but not completion of colonoscopy. 0.7% of controls completed colonoscopy (95% CI: 0.2–1.2%) compared to 1.2% in the incentive group (95% CI: 0.5–1.9%). *P* =.25.
Mehta et al., 2020b	United States	FIT	Incentive	281	Arm 1: text message alone. Arm 2: text message with lottery incentive- 1in5 chance of $100 after FIT completion.	FIT completion rate:Arm 1: 12.1% (95%CI: 6.7–17.5%)Arm 2: 12.1% (95%CI: 6.7–17.5%) No statistical difference.
(Raine et al., 2016)	England, UK	gFOBT	Norm (injunctive)	265,434	Control: standard invitation. Arm 1: GP endorsed letter, endorsement in the form of a banner.	Control uptake:58.2%, arm 1 uptake: 57.5%.After adjustment, GPE increased odds of uptake (OR = 1.07, 95% CI 1.04–1.10).
(Stoffel et al., 2019)	Spain	FOBT	Norm	5,077	Control: brochure, pharmacy list, invitation & reminder. Arm 1: intention prompt: control plus intention prompt. Arm 2: norms condition: control plus intention prompt plus invitation with injunctive norm. Arm 3: benefit condition: control, plus intention prompt, plus invitation with benefit condition.	8 percentage point increase in screening as a result of reminder letter.None of the interventions influenced participation: prompt OR 0.85 (95% CI: 0.75–1.08), social norms OR 1.00 (95% CI: 0.83–1.20), benefit OR 0.90 (0.75–1.08). Only significant difference was for new invitees, participation rates decreased from 34.9% to 24.2% when the invitation mentioned the importance of regular participation.
Church et al., 2004	United States	FOBT	Default	1,451	Control: questionnaire only. Arm 1: mailed FOBT with reminders. Arm 2: mailed FOBT without reminders.	Direct mailing of FOBT combined with reminders promoted increases in adherence. 1 year rate changes in absolute percentage for self-report FOBT adherence were:Control: 1.5% (95% CI:2.9%-5.9%)Arm 1: 16.9% (95% CI: 11.5%–22.3%) Arm 2: 23.2% (95% CI: 17.2%-29.3%).
MACS group, 2006 (MACS, 2006)	Australia	FOBT, colonoscopy, CTC, FS	Default	1,333	Arm 1: FOBT. Arm 2: FOBT & FS. Arm 3: CTC. Arm 4: colonoscopy. Arm 5: choice of 4 tests, mailed FOBT. Arm 6: choice of 4 tests, phone request FOBT.	Choice of test did not increase participation. Arm 5 participation 18.6% (*P* =.03) & arm 6 participation 22.7% (*P* =.3).
Inadomi et al., 2012	United States	FOBT, colonoscopy	Default	997	Arm 1: recommended FOBT. Arm 2: recommended colonoscopy. Arm 3: choice of FOBT or colonoscopy.	Colonoscopy arm completion (38.2%) was significantly lower than FOBT (67.2% *P* <.001) or choice arm (68.8% *P* <.001). Difference between FOBT & choice arms was non-significant (*P* =.64).
Van Roosbroeck, Hoeck & Van Hal, 2012	Belgium	iFOBT (FIT)	Default	19,542	Control: invitation letter with instructions to collect kit from GP. Arm 1: direct invitation letter with kit. Cross-over intervention after 6 weeks.	Participation by mail 52.3%, participation by GP 27.7%.Significant difference of 24.6% (95CI: 26.7–28.6% *P* <.001).Effect stronger after controlling for age, sex & area – participation almost 3 times higher for those invited by mail compared to GP, OR 2.96 (95%CI: 2.78–3.14, *P* <.001).
(Tinmouth et al., 2015)	Canada	gFOBT	Default	3,594	Control: mailed invitation from PCP. Arm 1: mailed kit & invitation from PCP.	Uptake > twice as high in arm 1: Intervention 20.1%, control 9.6%., absolute difference 10.5% (95% CI: 7.5–13.4%. *P* <.0001).
Mehta et al., 2018	United States	FIT	Default	314	Arm 1: opt-in text message. Arm 2: opt-out text message.	Opt-out improved participation, absolute difference in FIT completion rate: 19.5% (95% CI: 10.9–27.9%, *P* <.001).
Huf et al., 2021	United States	FIT	Default	440	Control: usual text message reminder. Arm 1: pre-alert text offering opt-out of receiving FIT kit & <3 behaviourally informed reminders.	Arm 1: 19.6% participation vs control: 2.3%, absolute increase of 17.3% (*P* <.001).FIT kit return: arm 1: 19.1% vs control: 1.4% control, 17.7% absolute increase (*P* <.001).
O’Carroll et al., 2015	Scotland, UK	FOBT	Salience	59,366	Control: pre-notification letter 2 weeks before kit, no questionnaire. Arm 1: HLOC: pre-notification, HLOC scale, ICK & perceived benefit items, two items indicating intention to return kit. Arm 2: AR: pre-notification, HLOC/ICK/perceived benefit/intentions questionnaire & two additional AR questions.	No overall difference between treatment groups on FOBT uptake: Control: 57.3% HLOC: 56.9% AR: 57.4%
Hagoel et al., 2016	Israel	FOBT	Salience	48,091	Control: no intervention. Arm 1: interrogative condition. Arm 2: interrogative plus social context condition. Arm 3: non-interrogative condition. Arm 4: non-interrogative plus social context.	Arm 1 (9.8%) & arm 2 (10.3%) had higher participation rates vs control (8.5%). After controlling for age, SES, and gender, the question mode (interrogative) had an OR of 1.07 (95% CI = 1.004. 1.150; P =.038). Social context had no significant effect. QBE modestly effective in CRC screening: higher in arm 1 than the other 3 groups.
(Raine et al., 2016)	England, UK	gFOBT	Salience	168,480	Control: usual reminder. Arm 1: enhanced reminder banner and re-statement of screening offer.	0.7 percentage point higher participation rate in arm 1 (25.8%) vs control (25.1%). Not significant in univariate analysis, but was significant after adjusting for age, sex, hub, and screening episode type, *P* =.001.
Mehta et al., 2019a (Mehta et al., 2019)	United States	FIT, colonoscopy	Salience	438	Arm 1: direct phone no. for scheduling colonoscopy only. Arm 2: sequential choice: phone no. to schedule colonoscopy & mailed kit if no response within 4 weeks. Arm 3: active choice: phone no. for colonoscopy & FIT offered at the same time.	4-month completion rates: (not significantly different) Colonoscopy only – 14.4% Sequential choice – 17.1% Active choice – 19.9% 6 months: (not significantly different) Colonoscopy only – 18.5% Sequential choice – 19.2% Active choice – 23.3%
Bakr et al., 2020 (Bakr et al., 2020 Jan)	United States	FIT, FS, colonoscopy, CTC, FIT-DNA	Salience	1,882	Control: standard text-based letter to encourage participation. Arm 1: letter leveraging social psychology & BE principles.	Uptake significantly higher in the arm 1 at each time interval. 1 month: 9.9% uptake in control, 15.2% in arm 1 (*P* <.001). 6 months: 19.5% in control, 24.1% arm 1 (*P* <.001).
Stoffel et al., 2021a (Stoffel et al., 2021 Mar)	Malta	FIT	Salience	8,349	Control: invitation letter with standard opt-in strategy. Arm 1: enhanced active choice: modified opt-in strategy.	Arm 1 did not significantly increase kit acceptance: OR adj 1.07 (95% CI: 0.98–1.18, p = 0.141).Arm 1 did not significantly increase participation: OR adj 1.03 (95% CI: 0.94–1.13, p = 0.565).
Stoffel et al., 2021b (Stoffel et al., 2021 Dec)	Cyprus	FIT	Salience	RCT 1: 3,131 RCT 2: 2,791	RCT 1: Control: usual invitation letter Arm 1: social responsibility message Arm 2: anticipated regret message Arm 3: account effect message Arm 4: benefit of early detection message Arm 5: scarcity message Arm 6: additional message on social norms RCT 2: Control: usual invitation letter Arm 1: social responsibility message	RCT 1: No significant differences between control and experimental conditions for the overall sample or men (all *P* >.05).Social responsibility message did increase uptake rates among women (25.6% vs. 17.1%, adj OR 1.67; 95% CI 1.05–2.66, *P* =.031). RCT 2: No impact on overall or uptake of men and women separately (all *P* >.05).
Coronado et al., 2020 (Coronado et al., 2020 Jul 30)	United States	FIT	Primer	2,825	Arm 1: text notification 1–2 days before anticipated receipt of mailed FIT. Arm 2: live phone call notification < 3 weeks before receipt of FIT.	ITT: FIT completion rates higher in arm 2 vs arm 1 (percentage point difference, 3.3%; 95% CI, 0.4%–6.2%). PP: between-group differences increased to 7.3% points (95% CI, 3.6%–11.0%) for participants reached by the text message, live call or voice message.PP rate increased to 14.9 percentage points (95% CI, 9.6%-20.1%) for participants reached by the text message or live call. Live phone calls outperformed text messages.

### 3.3 Risk of bias

The results of the RoB 2 analysis are presented in Table 5 (the completed worksheets for each study are available from Open Science Framework: https://osf.io/tqsmc/). The majority of studies had some bias (N = 16, 53%), which was largely due to missing data or contamination of the intervention groups.

### 3.4 Results stratified by MINDSPACE component

The most frequently tested BE intervention was incentives. Eleven studies (37%) evaluated the effectiveness of different financial incentives, such as cash payments and lotteries. Over half of these studies (N = 6, 54.5%) found that incentives significantly increased participation in CRC screening (Nisa et al., 2019, Kullgren et al., 2014, Slater et al., 2018, Lieberman et al., 2019, Green et al., 2019, Mehta et al., 2017), while the reminder (N = 5, 45.5%) reported no statistically significant effect (Dacus et al., 2018, Mehta et al., 2019a, Mehta et al., 2020a, Mehta et al., 2020b, Gupta et al., 2016). Cash payments, conditional on participating in the screening programme, were tested in eight studies (Slater et al., 2018, Mehta et al., 2019a, Lieberman et al., 2019, Green et al., 2019, Gupta et al., 2016, Nisa et al., 2019, Kullgren et al., 2014, Dacus et al., 2018), and only few found a positive statistically significant effect on screening participation (N = 3; 32,43,44; see Table 7). Lotteries with cash prices were tested less frequently (N = 5; 23,39,41,42,44) and only two showed statistically significant positive effects (Kullgren et al., 2014, Green et al., 2019). There were no differences in the risk of bias scores between studies that found a positive association and those that found no association (see Table 8).

**Table 7:** Types of financial incentives tested in studies.

**Type**	**Payment**	**Amount**	**Screening**	**Significant positive effect**	**No effect**
Lump sum	Conditional	$5	FOBT, FIT		Gupta et al., 2016; (Kullgren et al., 2014); Lieberman et al.; 2019
		$10	FOBT, FIT, colonoscopy	Green et al., 2019; Lieberman et al., 2019	Green et al., 2019; Gupta et al., 2016; (Kullgren et al., 2014); Lieberman et al.; 2019; Mehta et al., 2019b
		$20	Colonoscopy	Slater et al., 2018	
		$25	FOBT, sigmoidoscopy, colonoscopy		Dacus et al., 2018
		$100	Colonoscopy	Mehta et al., 2017	
	Conditional (split)	€ 10	FOBT	Nisa et al., 2019	
	Unconditional	$10	FIT		Mehta et al., 2019b
		$25	Colonoscopy		Mehta et al., 2020a
Lottery	Conditional	$100 (20% probability)	FIT		Mehta et al., 2020b
		$100 (10% probability)	FIT		Mehta et al., 2019b
		$50 (10% probability)	FOBT, FIT, FS, colonoscopy	Green et al., 2019; (Kullgren et al., 2014)	
		$500 (1% probability)	FOBT		(Kullgren et al., 2014)

**Table 8:** Results stratified by MINDSPACE component.

**MINDSPACE Component:**	**Studies reporting a positive effect (N = 18):**				**Studies reporting no effect (N = 12):**				**Total RoB 2 score**
	**Low risk**	**Some concern**	**High risk**	**RoB 2 score**	**Low risk**	**Some concern**	**High risk**	**RoB 2 score**	
Messenger (N = 2)		1 (50%)	1 (50%)	3/6 (50%)				N/A	**3/6** **(50%)**
Incentive (N = 11)	4 (36%)	2 (18%)		16/18 (89%)	3 (27%)	2 (18%)		13/15 (87%)	**29/33** **(88%)**
Norms (N = 2)		1 (50%)		2/3 (67%)		1 (50%)		2/3 (67%)	**4/6** **(67%)**
Default (N = 7)	2 (29%)	2 (29%)	1 (14%)	11/15 (73%)		2 (29%)		4/6 (67%)	**15/21** **(71%)**
Salience (N = 7)		3 (43%)		6/9 (67%)	2 (29%)	2 (29%)		10/12 (83.3%)	**16/21** **(76%)**
Primer (N = 1)	1 (100%)			3/3 (100%)				N/A	**3/3** **(100%)**
**Subtotal (N = 30)**	**21/21**	**18/27**	**2/6**	**41/54** **(76%)**	**15/15**	**14/21**		**29/36** **(81%)**	**70/90 (78%)**

Note: Studies with low risk of bias were given 3 points, those with some concern 2 point and high-risk studies received 1 point. The RoB 2 score summarises the points of the overall studies and for those reporting a positive effect or no effect, separately, as well as by MINDSPACE component.

The next most frequently tested BE intervention was the default principle (N = 7, 23.3%). There, three studies (42.8%) tested opt-out approaches, two (28.6%) offered a choice between different screening tests and the remainder either reminder or pre-alert messages (N = 2, 28.6%). The majority of studies (N = 5, 71.4%) testing this style of intervention found a statistically significant positive effect on participation (Tinmouth et al., 2015, Van Roosbroeck et al., 2012, Mehta et al., 2018, Church et al., 2004, Huf et al., 2021), while the remainder (N = 2, 28.6%) found no statistically significant positive or negative effect on participation (Inadomi et al., 2012, MACS, 2006). Employing the default component using an opt-out approach with direct mailing of the screening test kit was always effective in increasing uptake (Tinmouth et al., 2015, Van Roosbroeck et al., 2012, Mehta et al., 2018). Similarly, pre-alert and reminder messages were also always found to be effective (Church et al., 2004, Huf et al., 2021). Offering choice, however, never increased uptake compared to recommending specific tests (Inadomi et al., 2012, Tinmouth et al., 2015, MACS, 2006). There was no difference in the risk of bias scores for those that found a positive effect and those that found no effect.

As with defaults, seven papers tested the effectiveness of salience on CRC screening participation. Three studies (42.8%) investigated messages from social psychology and BE (Raine et al., 2016, Stoffel et al., 2021a, Bakr et al., 2020), two studies (28.6%) alternative choice framings (Mehta et al., 2019b, Stoffel et al., 2021b) and two studies (28.6%) concepts from psychology, namely: anticipated regret and the question-behaviour effect (O’Carroll et al., 2015, Hagoel et al., 2016). Only three studies (42.8%), two on BE messages and one on the question-behaviour effect found a statistically significant positive effect (Raine et al., 2016, Bakr et al., 2020, Hagoel et al., 2016), with most studies (N = 4, 57%) finding no statistically significant positive or negative effect on participation (Stoffel et al., 2021a, Mehta et al., 2019b, O’Carroll et al., 2015, Stoffel et al., 2021b). Moreover, the risk of bias scores for studies that reported a positive effect, compared to those reporting no effect, were higher, indicating that the papers reporting no effect were of better quality.

Few interventions tested the effects of norms (N = 2, 7%) and results were divided equally between a significant positive effect for a study that employed an injunctive norm, stating that the GP endorses the screening programme and that participation is desired (Raine et al., 2016) and no significant positive or negative effect on behaviour for a study that used descriptive norms, communicating that the majority of people get screened (Stoffel et al., 2019). Both studies have some concern for biases.

The messenger component was also tested in two studies, both of which reported a statistically significant positive effect (Cole et al., 2002, Zajac et al., 2010). In both studies, the messenger component consisted of an invitation letter with the practice letterhead and signature of the GP or practice partner. The quality of the studies, however, was low as one study had risk of bias (Zajac et al., 2010) and the other raised some concerns (Cole et al., 2002).

One study tested the effectiveness of primers and found a significant positive effect (Coronado et al., 2020). The study had a low risk of bias and tested the effectiveness of pre-notification phone calls three weeks before the anticipated receipt of the test kit.

No studies testing the effectiveness of affect, commitment or ego were identified (see Table 8).

## 4 Discussion

### 4.1 Summary of main findings

This review sought to synthesise the available evidence from RCTs for using BE interventions to improve CRC screening participation. A total of 30 studies were identified, the majority of which were conducted in the USA and examined the effectiveness of BE interventions to promote the uptake of gFOBT and FIT screening. Importantly, most studies found that BE interventions resulted in a statistically significant increase in CRC screening participation suggesting that BE interventions have the potential to improve participation. The differences observed between studies that reported a positive effect on CRC screening uptake, compared to those that reported no effect, are unlikely to be attributable to bias, as the papers are of similar quality.

Default-based interventions had the best evidence to support their use in terms of the proportion that found a positive effect and a low risk of bias. Employing the default component using an opt-out approach with direct mailing of the screening test kit was more likely to be effective than recommending specific tests. Similarly, when using the messenger component, presenting the screening offer on practice letterhead and including the signature of a practice partner appears more effective than simply indicating GP support (Cole et al., 2002, Zajac et al., 2010). Incentives, meanwhile, had mixed evidence to support their use; evidence is particularly uncertain outside of the USA, where all but one study tested their effectiveness. Additionally, as US studies were not conducted as part of organised screening programmes, the generalisability of their findings may be limited for organised programmes. Although the types of interventions utilising the salience component were varied, active choice and anticipated regret interventions were seemingly less effective than use of other salience-informed mailings. Only a single study leveraged the primer component with pre-notification phone calls (and despite the fact that it was of low risk of bias), it is difficult to draw conclusions about the overall efficacy of priming to improve CRC screening participation.

The results of this review also suggest that the quality of the literature is mixed, with 60% of the included studies raising some degree of concerns for bias.

### 4.2 Comparisons with the previous literature.

The results of this review are consistent with the findings of studies examining the effectiveness of BE interventions to modify other behaviours. For example, the finding that defaults are effective at improving CRC screening participation are consistent with studies assessing their effectiveness to improve organ donation (Johnson and Goldstein, 2003). The results of this review are also consistent with those of a previous review of interventions to promote cancer screening participation, which also found that GP-endorsement (messenger) and mailing kits with the invitation (default) were effective. Finally, the results of this review are also consistent with a recent review of the evidence for use of incentives to improve participation in CRC screening, specifically (Facciorusso et al., 2021). Not only this, but the present review identified all the papers included in the incentives review (as well as several additional ones), suggesting that the rapid search strategy used was effective.

### 4.3 Implications for policy and future research.

The finding that most BE interventions have no effect or a positive effect on CRC screening participation suggests that they can be adopted into formal screening programmes with minimal risk to adversely affecting participation. Moreover, as recent cost-effectiveness analyses have shown that BE interventions can be cost-effective in increasing vaccination and improving antibiotic prescribing for acute respiratory infections (Benartzi et al., 2017, Gong et al., 2019). Most interventions (aside from those leveraging financial incentives) involve relatively low costs, so that they are unlikely to significantly influence the finances of the impact screening programmes (RoB 2, 2021), further justifying their use in line with APEASE criteria.

The results of this review do, however, suggest that further research into specific MINDSPACE components is required to determine their effectiveness in promoting participation in CRC screening, namely: norms, messenger, and primer-based interventions. In addition, the results of this review suggest that further research is needed to determine the effectiveness of BE interventions in a wider range of settings and contexts. For example, no studies conducted in Asian countries were identified, despite the fact that many of these countries do offer organised screening programmes for CRC and operate differently to the USA (Chiu et al., 2017). Finally, the present review suggests that further high quality research is needed in this area, as most studies had some or high risk of bias.

## 5 Strengths and limitations

This review has several strengths. First, no restrictions were placed in terms of when studies were published, meaning no literature was excluded on this basis. Second, the review focussed solely on CRC screening, making the results highly specific to CRC screening behaviours. Finally, a relatively high number of papers were included, considering BE in cancer screening is a relatively new field of research. Note that alternative empirical studies, such as online experiments, were not included in this study as they do not measure real behaviour (Stoffel et al., 2019, Stoffel et al., 2019, von Wagner et al., 2019, Stoffel et al., 2019, Stoffel et al., 2020).

This review also has several limitations. Most importantly, the search strategy used was not comprehensive; it was limited to peer-reviewed articles available on PubMed, PsycInfo and EconLit. Furthermore, several search terms were omitted, due to zero articles being identified by the final search. As such, it is possible that our review did not include several relevant studies. This is a common limitation with rapid reviews, one which is often accepted in favour of reviewing the literature in a shorter period, usually because the time and resources required for a fully comprehensive systematic review are not available. Additionally, the search did not include grey literature and non-English studies, which may have led to a selection bias with an over-representation of studies with statistically significant findings. While the review only included studies that were conducted in organised screening settings, BE interventions were often tested in only single countries (e.g. almost all incentive studies were conducted in the USA), thus generalisability may be limited outside of these countries.

Finally, stratification using the MINDSPACE framework may be an oversimplification of BE interventions, as the framework leaves little room to assess the impact of overlapping characteristics on screening behaviours, and it is difficult to disentangle individual effects.

## 6 Conclusion

This review shows that the effectiveness of BE interventions to promote CRC screening participation is mixed. The majority of studies have had a positive effect on screening participation, but a considerable proportion have found no effect. Several areas remain in need of future research, including investigation into BE interventions in programmes outside of the USA. Overall, BE remains a promising field of interest in relation to influencing CRC screening behaviours.

## Declaration of Competing Interest

The authors declare that they have no known competing financial interests or personal relationships that could have appeared to influence the work reported in this paper.
